# Increasing Physical Activity Efficiently: An Experimental Pilot Study of a Website and Mobile Phone Intervention

**DOI:** 10.1155/2014/746232

**Published:** 2014-05-22

**Authors:** Kjærsti Thorsteinsen, Joar Vittersø, Gunnvald Bendix Svendsen

**Affiliations:** ^1^Tromsø Telemedicine Laboratory, Department of Psychology, University of Tromsø, 9037 Tromsø, Norway; ^2^Department of Psychology, University of Tromsø, 9037 Tromsø, Norway; ^3^Brand Management, Telenor ASA, Snarøyveien 30, 1331 Fornebu, Norway

## Abstract

The main objective of this pilot study was to test the effectiveness of an online, interactive physical activity intervention that also incorporated gaming components. The intervention design included an activity planner, progress monitoring, and gamification components and used SMS text as a secondary delivery channel and feedback to improve engagement in the intervention content. Healthy adults (*n* = 21) recruited through ads in local newspapers (age 35–73) were randomized to the intervention or the control condition. Both groups reported physical activity using daily report forms in four registration weeks during the three-month study: only the experiment condition received access to the intervention. Analyses showed that the intervention group had significantly more minutes of physical activity in weeks five and nine. We also found a difference in the intensity of exercise in week five. Although the intervention group reported more minutes of physical activity at higher intensity levels, we were not able to find a significant effect at the end of the study period. In conclusion, this study adds to the research on the effectiveness of using the Internet and SMS text messages for delivering physical activity interventions and supports gamification as a viable intervention tool.

## 1. Introduction

Being physically active is one of the most important predictors of mental and physical health [1]. In order to benefit from the positive effects of physical activity and avoid the negative effects of physical inactivity, it is important that we adhere to the World Health Organization's (WHO) recommendations for physical activity [2]. One problem is that some of us lack knowledge of the recommendations to be physically active for 30 minutes at a moderate intensity on most, preferably all, days of the week [3]. A more complex and tangible problem is that most of us fail to do so, even though we are fully aware of the consequences [3–5]. Researchers and clinicians working with behavior change have acknowledged that there is a gap between knowing what we should do and actually doing it. Thus, interventions to help people change their undesirable behavior and embrace more healthy habits have been developed.

Up-to-date intervention researchers utilize new opportunities for delivery of health promotion interventions brought on by advances in technology. Computer-tailoring an intervention and disseminating it through the Internet using a website, by e-mail and short mobile service (SMS) text messages, is a promising health education strategy [6–12]. This way people can get access to individually tailored advice, regardless of geographical and temporal barriers, and without the cost of a one-by-one consultation [13–15]. Still, one recent review found that the most effective interventions were delivered face to face [16]. One of the main challenges at this time is to maintain use of the interventions over time, as many studies of online computer-tailored interventions report an initial effect that wears off [6, 13, 17–21]. The decreased effect over time could be a result of decreased exposure to the intervention because of static content and boredom when the novelty of the system wears off [17, 21]. Thus, planning interventions that can maintain engagement over time is important. In a prestudy, potential consumers' intention to use a physical activity intervention (delivered online through the Internet and mobile phone) was influenced by perceived usefulness and expecting to have fun while using the system [22]. Ease of use, social support, and interactive features have also been reported to be important for usage of such systems [23, 24]. Naturally, previous studies have found that only active users of physical activity interventions improve their physical activity levels [25, 26]. Further developing and incorporating easy, social, and fun to use components in physical activity interventions is therefore an important way to increase usage and intervention exposure, consequently potentially produce more effective interventions.

One way to make interventions more fun is to add a game dimension. Gaming elements in nongame contexts of behavior change are an increasingly popular strategy to solve the adherence problem in health-related behavior [27, 28]. In what is known as gamification [29], interactivity is obligatory and making it easy and fun to use is part of the design process. Social components can also be integrated. Gaming components that require the participant to log in and register activity in order to make advancement in the game provide an extra incentive to use the intervention. However, so far there are not many interventions that utilize this opportunity.

Using SMS as a secondary delivery method has been shown to increase the effectiveness of website interventions [10, 30, 31]. Interventions using SMS as the primary delivery channel have also shown promising results for health care interventions [11]. In 2013 there were almost as many mobile-cellular subscriptions as people in the world [32]. Because people bring their mobile phones anywhere, they are perfect for instantaneous delivery of short intervention messages directly to individuals at any time, place, or setting. Also, they provide a means to give real-time feedback and just-in-time support for behavior change that could help the effectiveness of the intervention through improved adherence [33]. Although SMS messaging has been shown to be a viable intervention tool, there is a need for more research [34–36].

The present study reports a pilot test of an interactive, computer-tailored website physical activity intervention, with additional SMS text messages. The intervention was named Lifestyle Tool and is described further in the Method section. The goal of Lifestyle Tool was to motivate users of the program to incorporate physical activity in their daily routine, as doing so increases the chances of maintaining exercise over time [37]. The system was designed to be easy and fun to use and it incorporated social and individual gaming components to increase motivation and engagement. In order to closely monitor when the effect of the intervention occurred and how its influence projected over time, we wanted to assess participants' physical activity regularly. A three-month randomized controlled pilot trial with regular registration periods was conducted to compare the efficacy of the intervention on improving levels of physical activity in overall healthy adults with a control group. We hypothesized that participants randomized to the intervention condition would improve in physical activity more than the control group but that the effect would be greatest on the first measurement after implementation.

## 2. Materials and Method

### 2.1. Participants and Procedure

**Figure 1:**
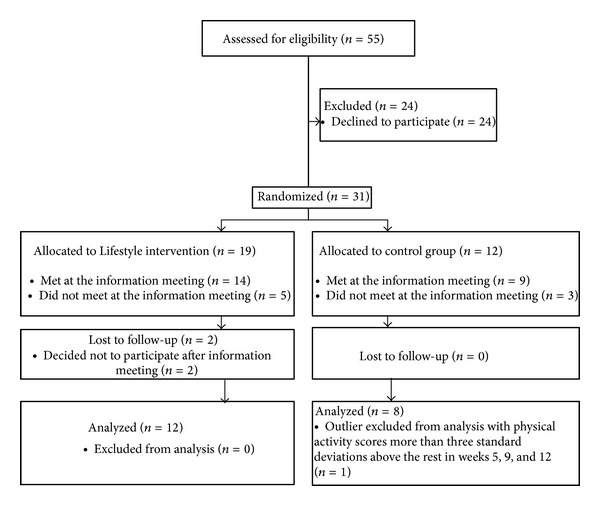
Participant flow diagram.

Participants were recruited through an advertisement in local newspapers in the town of Tromsø, Norway, in December 2008, which inspired 55 individuals to make initial contact and request more information about the study. The potential participants received additional information over e-mail and were asked to respond to a small set of initial questions such as age and gender. Additionally, using a short stages-of-change questionnaire [38], they reported on their level of physical activity. Inclusion criteria were access to the Internet and owning a mobile phone in addition to general good health (e.g., not having any known medical diseases). Of the 55 individuals that first made contact, 31 agreed to participate in the study and were invited to an information meeting in January 2009. The 31 participants were randomly assigned to either an experimental condition (the Lifestyle group) or a control condition. To ensure equal representation of age, gender, and physical activity level in the two groups the answers to the initial questions over e-mail were used to stratify the participants in sets. Based on the stages-of-change questionnaire [38] participants were given a contemplation score; precontemplation, contemplation, preparation, action, or maintenance. Participants belonging to precontemplation, contemplation, and preparation stages were put in one set (passive, *N* = 14); participants belonging to the action and maintenance stages were put in the other set (active, *N* = 17). Two sets were also created based on age (below/above average) and gender (male/female). Members from each set were then randomly assigned to the two experimental groups, 19 to the Lifestyle group, 12 to the control group. A flow chart of the grouping process following the Consort guidelines [39] is provided in Figure 1.

Participants were blinded to group allocation and not aware of the existence of another group. Both groups received identical envelopes by mail consisting of a consent form and daily report forms of physical activity which they were asked to complete one week before the initial information meeting. A total of 23 participants met at the information meeting and handed in consent and baseline (week 1) daily report forms.

Two participants from the Lifestyle group dropped out after the meeting. Thus, the final sample consisted of 21 participants—11 men and 10 women—who ranged in age from 35 to 73 years (*M* = 55.3, SD = 11.2). The Lifestyle group consisted of 12 participants; the control group consisted of 9 participants. However, one of the participants in the control group was far more active than the others (with physical activity scores more than three standard deviations above the rest) in weeks 5, 9, and 13. He was identified as an outlier and removed from subsequent analyses. This left 8 participants in the control group and 12 participants in the Lifestyle group.

The small sample size puts serious limitations to the statistical power of our study. For example, using 2-sided test and 5% significance level showed that we had 54% power to reject the null hypothesis of equal means if the population mean difference is 1.0 with a standard deviation for both groups of 1.0 (based on a calculation using the PASS 12 software) [40].

At the initial meetings (that were held separately for the two groups) both groups received general information about the study. In addition, the Lifestyle group was given information about—and basic training in using—Lifestyle Tool, and they were asked to create a personal account on the Lifestyle Tool website the next day. All of the participants in the Lifestyle group made an account on the Lifestyle portal within three days from the initial meeting.

**Figure 2:**
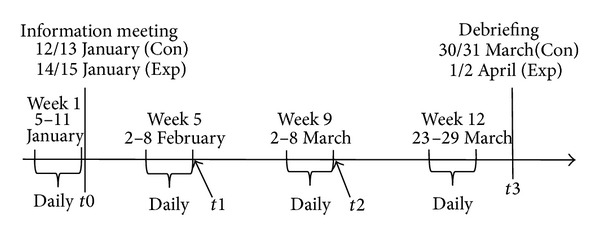
Study timeline. Registration of physical activity every night of weeks 1, 5, 9, and 12.

Participants in both groups completed daily report forms of physical activity (described in Section 2.3) every night of the week, roughly every four weeks over three months. They also filled out a set of questionnaires with 61–79 items at the information meeting (*t*0), at the end of registration weeks five and nine (*t*1 and* t*2) and at the debriefing (*t*3; see study timeline in Figure 2). The answers to these questionnaires are not included in any of the analyses, thus they are not further described in this paper. Participants in the Lifestyle group were encouraged to use the Lifestyle Tool intervention during the three-month period the study lasted. As reward for their participation, all participants took part in a lottery for three gift certificates of 5000 Norwegian Kroner (approximately 900$) at the end of the study.

### 2.2. Intervention

The intervention Lifestyle Tool consisted of a rule-based website designed to help people plan and monitor their physical activity in order to become more physically active. Participants in the Lifestyle group created a user-account on the Lifestyle Tool portal which gave them access to their personal page. On the first log-in they were asked to fill out the behavioral regulation in exercise questionnaire (BREQ-2: [41, 42]) which assessed motivational level for physical activity. When logged on the personal page of Lifestyle Tool, participants had access to a physical activity calendar where they could fill in and plan their physical activities. The program made a graphical representation of the accomplished physical activity each week, with the recommended guidelines [2] plotted in the same chart. This gave participants an opportunity to monitor their own level of physical activity in relation to the guidelines (self-monitoring) and set specific goals [43, 44]. Participants also received text messages by SMS from a message library with educational information of the benefits of being physically active and the potential harmful risks of being inactive, in addition to concrete tips on what to do. The messages were tailored using personalization and adaption [45]. Messages were personalized by referring to the participants by their first name, which is thought to activate the process of self-referent encoding (superior memory of information with reference to the self). The information content was individually adapted to match participants age and gender, and the number of messages each participant received each week was based on their motivational level for physical activity (measured with the BREQ-2 questionnaire).

To make Lifestyle Tool more interactive and fun to use, we included a game-component in which participants received points for completed activities. When participants had registered an activity, the system generated a reminder-text by SMS 30 minutes before the activity should start according to the plan. Then, 30 minutes after the activity ended the system generated a new SMS text asking the following. (1) To which degree did you complete the activity according to the plan? (2) How exhausting was it? And (3) how pleased are you with what you have accomplished? The responses to the first two questions in addition to the length of the activity gave the basis for how many points the participant received for a given activity. The answers to these questions also determined the feedback-message that was sent automatically, designed to positively reinforce complying behavior or help improve compliance in the future. Accumulated points were visible on the participants' personal site in addition to a hierarchical category status level. The status level was based on how many points a participant had, ranging from novice to enthusiast. By earning points the participants went up the ladder.

Lifestyle Tool also included two gaming components designed to increase the motivation for being physically active and helping participants to set goals: social contract and competition. The social contract component consisted of an agreement between two or more participants. When participants accepted a contract proposal they committed to complete all of their planned physical activities within a period of time (the time-window was determined by the initiator of the agreement). If they were successful in adhering to the agreement participants received bonus-points (50% of the points they earned in this period were added to the regular points). No bonus points were handed out to participants who violated the contract. The competition component had two alternatives. Participants could initiate a competition where the winner was (a) the first one to be *xx* minutes physically active, or (b) the most physically active (most minutes of physical activity) in a period of time (set by the initiator). Also here, successful participants received bonus points (75% extra for first place, 50% extra for second place, and 25% extra for third place). In addition, they received medals for first (gold), second (silver), and third (bronze) places. Medals won were visible on the participants' personal page. The Lifestyle group participants received messages when they were invited to join a competition or engage in a social contract by SMS text and under “Messages” on their personal page in Lifestyle.

### 2.3. Measures

The outcome of primary interest in the study was change in physical activity behavior after the intervention was implemented. Daily report forms were used to assess physical activity. Participants reported what kind of physical activity they had completed that day (example given “walked fast to work”), the duration in minutes they engaged in this activity, and how strenuous each activity was using Borg's ratings of perceived exertion scale (RPE-scale; [46]) for a maximum of five activities each day. Borg's RPE scale ranges from 6 (no exertion at all) to 20 (maximal exertion). The scale is designed in such a way that approximate pulse can be found by multiplying the reported number with ten (e.g., if you score an activity as 8, your pulse during this activity would be around 80). It measures perceived exertion, which is the heaviness and strain experienced subjectively in physical activity, and is widely used as a measure of perceived physical exertion [47]. Using daily report forms constitutes a sort of day reconstruction method which relies on short recall periods. This method has the benefit of reducing errors and biases of recall [48]. One participant recorded car driving and shopping as exercise, while another recorded working. These activities were removed before further analyses were conducted. The daily report forms were completed every night of study weeks 1, 5, 9, and 12. Physical activity in minutes was added together to make a sum score for the weekly exercise minutes. The reported Borg scores for each activity were averaged to compute the average Borg score of each week. In addition, physical activity in minutes was combined with their respective Borg score (that is Borg *x* minutes) to compute an effectual physical activity score that was averaged.

### 2.4. Statistical Analyses

Data analyses were performed with SPSS for Windows (version 21, IBM corp.). The difference in physical activity minutes, Borg, and effectual physical activity between the control and the Lifestyle group in weeks 5, 9, and 12 was evaluated using an analysis of variance with a covariate (ANCOVA). Week 1 measures were included as a covariate in the model to control for the effect of initial physical activity. In all tests, differences were considered statistically significant if the *P* value was less than 0.05.

## 3. Results

The Lifestyle group participants registered 1046 activities in the physical activity (PA) planner. All of the participants used the PA planner, with a range from 31 to 178 activities for each of the participants, *M* = 87.17, SD = 44.24. All of the Lifestyle group participants also tried out the contest component and eight of the twelve participants participated in at least one social contract.

The two groups started out with the same physical activity minute in the first measurement week (baseline): *M* = 473.83, SD = 237.95 for the Lifestyle group and *M* = 485.50, SD = 221.40 for the Control group, *F*(1,18) = 0.030, *P* = 0.913. The Borg intensity level was also equivalent for the two groups at this time point: *M* = 12.53, SD = 1.85 for the Lifestyle group and *M* = 12.41, SD = 0.85 for the control group, *F*(1,18) = 0.030, *P* = 0.865. After the onset of the intervention the Lifestyle group performed consistently more physical activity, at a higher intensity, than the control group. However, the differences between the two groups were not always significant (see Table 1).

As can be seen from Table 1, the Lifestyle group reported significantly more minutes of physical activity than the control group in week 5 (Lifestyle *M* = 576.67, SD = 235.29 versus control group *M* = 413.13, SD = 224.42, *F*(1,17) = 4.739, *P* = 0.044) and week 9 (Lifestyle group *M* = 660.25, SD = 359.60 versus control group *M* = 377.25, SD = 167.39, *F*(1,17) = 4.510, *P* = 0.049). In week 12 the difference between the two groups was no longer significant. In week 5 (the first measurement week after the intervention started), the Lifestyle group (*M* = 13.52, SD = 1.98) performed physical activity at a significantly higher Borg intensity level than the control group (*M* = 12.01, SD = 2.00), *F*(1,16) = 5.208, *P* = 0.037. In the other weeks there were no significant differences for the intensity of the exercise. For effectual physical activity (Borg ∗ minutes) there was a significant difference between the Lifestyle group (*M* = 247.53, SD = 137.65) and the control group (*M* = 114.95, SD = 72.12) only in week 9, *F*(1,17) = 6.115, *P* = .024.

## 4. Discussion

The present pilot study suggests that including gaming elements and SMS-text in an interactive, computer-tailored physical activity intervention is useful. All of the participants in the Lifestyle group registered physical activity in the online planner and received points when reporting back afterwards using SMS text. All of them also joined one or more competitions and the majority joined at least one social contract.

An initial boost in minutes of physical activity was observed. In week 5 the participants who received the intervention performed significantly more physical activity minutes than the control group and the effect was sustained in week 9. However, the effect was gone in the last registration week of the study period as observed elsewhere [21]. Corroborating this, newer studies and reviews continue to report declining effects over time [19, 49, 50]. The initial effect is promising. Despite few participants and high activity level at baseline (both groups exercised more than the recommended minimum amount in registration week 1), there was a significant increase in physical activity for the intervention group compared with the control group in weeks 5 and 9. By three months, although the Lifestyle group still performed more minutes of physical activity than the control group, the effect was not significant. The Lifestyle group also consistently reported performing more strenuous physical activity than the control group, and this difference was significant in week 5. In week 9 the combination of Borg and minutes was higher for participants in the Lifestyle group compared to the control group: participants receiving the intervention completed more physical activity at a higher intensity level than participants in the control group at this time. The intervention seems to have encouraged participant to do more moderate and vigorous exercise which is positive considering the dose-response relation between physical activity and health benefits [1].

Our results are supplement to the growing evidence supporting the effectiveness of online tailored physical activities for increasing physical activity [18–20, 51] and suggest using gamification and SMS texts as viable tools that need further investigation [52, 53].

This pilot study has some limitations. First, as this was an initial study with a small number of participants there are constraints regarding generalizing the results. However, finding a significant effect of the intervention even with only a small sample size attests to the robustness of the reported effects, at least for the population from which our sample was recruited. Because of the small sample size we were not able to analyze which components of the interventions were most effective in increasing physical activity. Feedback from participants suggested that the gaming components, especially the individual competition (advancing to the next status level for physical activity) and social competition (winning a competition), were highly motivating. Future studies with larger populations should identify which components are more effective.

Second, recruiting was done by self-selection; interested participants responded to an ad in the paper. This leaves us with highly motivated participants, and perhaps not those that need it the most [54]. Both the control and the Lifestyle group exercised more than the recommended guidelines at baseline (week 1) even though about half reported being passive three weeks before the baseline measure was completed. One possible explanation could be that the study started in January, which for some could mean a New Year resolution to exercise more. Also it could be a measurement effect; the daily report forms of physical activity (which both groups completed) would trigger awareness of own physical activity level which is an important starting point for behavior change [55–57]. However, even though the baseline measure was high, participants using Lifestyle Tool increased their physical activity and maintained a higher physical activity level than the control group throughout the study period.

Third, in this study, we used self-reports to measure physical activity. Although using daily report forms would reduce memory bias and thus yield more accurate reports than retrospective questions, participants still could report more physical activity than they actually completed (e.g., social bias). Measuring physical activity objectively; for example, using novel sensing technology already available in cell phones should be a goal for future studies.

In conclusion, using gamification in physical activity interventions is a fun and engaging way to help people achieve behavior change. The effect seems to drop off after some weeks though. Further development of gaming components in combination with using the mobile phone to stay connected with participants could help solve the adherence problem in intervention research. However, more research is needed in order to identify which components are more effective. The new technology increases opportunities to reach people where they are and when they need it. The entrance of smart phones allows further sophistication of interventions for health promotion and disease prevention; the challenge is to utilize the available technology optimally [26, 58]. However, text-messaging will likely continue to be used [59], especially in less developed countries.

## Figures and Tables

**Table 1:** Analyses of covariance for means in minutes exercising, Borg, and minutes ∗ Borg between the Lifestyle and the control group for the four measurement weeks. Significant (*P* < 0.05)  *F*'s in Bold.

	Minutes					Borg					Borg ∗ Minutes				
	Lifestyle		Control			Lifestyle		Control			Lifestyle		Control		
	M	CI	M	CI	*F*	M	CI	M	CI	*F*	M	CI	M	CI	*F*
	(SD)		(SD)			(SD)		(SD)			(SD)		(SD)		
Week 1	473.83	322.65–625.02	485.50	300.40–670.60	0.012	12.53	11.35–13.71	12.41	11.79–13.12	0.030	159.36	112.79–205.93	176.64	105.08–248.20	0.234
	(237.95)		(221.40)			(1.85)		(0.85)			(73.29)		(85.60)		
Week 5	576.67	427.17–726.16	413.13	225.51–600.74	**4.739**	13.52	12.19–14.85	12.01	10.34–13.69	**5.208**	196.84	127.57–266.10	150.44	69.11–231.77	1.231
	(235.29)		(224.42)			(1.98)		(2.00)			(109.01)		(196.84)		
Week 9	660.25	431.77–888.73	377.25	237.31–517.19	**4.510**	13.25	12.07–14.43	11.95	10.51–13.39	3.213	247.53	160.07–334.99	114.95	54.65–175.24	**6.115**
	(359.60)		(167.39)			(1.85)		(1.55)			(137.65)		(72.12)		
Week 12	574.42	298.20–850.64	501.88	307.16–696.59	0.264	13.34	11.85–14.83	12.75	11.85–13.65	1.731	210.69	111.49–309.90	178.69	103.82–253.56	0.616
	(434.74)		(232.90)			(2.22)		(1.08)			(156.13)		(89.56)		

Note: week 1 measures of minutes, Borg, and Borg ∗ minutes are included as a covariate in the analyses for week 5, 9, and 12.
